# Shoulder Range of Motion Measurement Using Inertial Measurement Unit—Validation with a Robot Arm

**DOI:** 10.3390/s23125364

**Published:** 2023-06-06

**Authors:** Martyna Białecka, Kacper Gruszczyński, Paweł Cisowski, Jakub Kaszyński, Cezary Baka, Przemysław Lubiatowski

**Affiliations:** 1 Rehasport Clinic, Gorecka 30, 60-201 Poznan, Poland; pawel.cisowski@rehasport.pl (P.C.); jakub.kaszynski@rehasport.pl (J.K.); cezary.baka@rehasport.pl (C.B.); p.lubiatowski@rehasport.pl (P.L.); 2 The Faculty of Mechanical Engineering, Institute of Applied Mechanics, Poznan University of Technology, 60-965 Poznan, Poland; 3 RSQ Technologies Company, 27 Grudnia 3, 61-737 Poznan, Poland; 4 Spine Disorders and Pediatric Orthopedics Department, Poznan University of Medical Sciences, 61-545 Poznan, Poland; 5 Orthopaedics, Traumatology and Hand Surgery Department, Poznan University of Medical Sciences, 28 Czerwca 1956, No. 135/147, 61-545 Poznan, Poland

**Keywords:** inertial measurement unit, sensor accuracy, validation, shoulder, range of motion

## Abstract

The invention of inertial measurement units allowed the construction of sensors suitable for human motion tracking that are more affordable than expensive optical motion capture systems, but there are a few factors influencing their accuracy, such as the calibration methods and the fusion algorithms used to translate sensor readings into angles. The main purpose of this study was to test the accuracy of a single RSQ Motion sensor in comparison to a highly precise industrial robot. The secondary objectives were to test how the type of sensor calibration affects its accuracy and whether the time and magnitude of the tested angle have an impact on the sensor’s accuracy. We performed sensor tests for nine repetitions of nine static angles made by the robot arm in eleven series. The chosen robot movements mimicked shoulder movements in a range of motion test (flexion, abduction, and rotation). The RSQ Motion sensor appeared to be very accurate, with a root-mean-square error below 0.15°. Furthermore, we found a moderate-to-strong correlation between the sensor error and the magnitude of the measured angle but only for the sensor calibrated with the gyroscope and accelerometer readings. Although the high accuracy of the RSQ Motion sensors was demonstrated in this paper, they require further study on human subjects and comparisons to the other devices known as the gold standards in orthopedics.

## 1. Introduction

Human movement tracking has been an interest of scientists for many years now, especially in orthopedics. Motion analysis in humans has been explored for a very long time, from visual assessment or using simple goniometers up to video recording and sophisticated motion capture systems. The main fields of this exploration were clinical applications and sport performance. In this paper, we focused on the shoulder, as it is the most mobile joint complex in the human body. Loss of range of motion (ROM) due to various disorders is a common clinical problem. Both surgical treatment and rehabilitation aim to gradually restore ROM. This process is usually closely monitored, and there is a certain follow-up time during therapy. Using sensors is an attractive option allowing for constant, self-applied, and remotely controlled recording. On the other hand, both professional and amateur sports performances are routinely measured by athletes and coaches. The precision of movement, movement patterns, and angular velocities are among the many values measured to monitor performance, detect weaknesses, and control improvement. Recent advances in technology allow us to measure joint angles, ROM, angular velocities, and accelerations with very high precision in natural environments or in activities of daily living. The gold standard in human motion tracking is optical motion capture (OMC) systems that use markers or sensors attached to human body landmarks in order to track the movement of the body segments in a restricted three-dimensional space (e.g., Vicon or OptiTrack) [1]. They are believed to have the highest accuracy for three-dimensional measurement of the available solutions on the market, but they are very expensive; the registered movement is restricted to the strictly defined laboratory space; and the data collection, processing, and interpretation are time-consuming.

The micro-electro-mechanical systems (MEMS) recently introduced into inertial measurement units (IMUs) allow the construction of sensors suitable for human motion tracking that are much more affordable, portable, and easy to use in clinical settings or everyday life [2]. These sensors usually include three main components, an accelerometer, a magnetometer, and a gyroscope, and use advanced fusion methods to translate the signal from its electronic components to information about the position of the limbs. The orientation of an IMU sensor is frequently represented by three angles, called roll, pitch, and yaw, which describe the rotation of the sensor with reference to one of the three mutually perpendicular sensor axes (x, y, and z, respectively). The roll and pitch angles, collectively named attitude, are usually the most accurate movements that can be measured with IMU sensors, while the estimation of the orientation angle in the yaw axis (heading movement) is vulnerable to magnetic disturbances from ferromagnetic materials and the environment [3]. Furthermore, the accuracy of an IMU sensor is highly influenced by the calibration method used by the sensor manufacturer, the software of the sensor in the form of an algorithm (sensor fusion technique), or the alignment error of the electronic components. Another common issue connected to IMU sensors is the increase in measurement error with time called ‘drift’, which is the effect of time-varying biases and noises connected to accelerometer and gyroscope readings. Algorithmic or hardware solutions usually compensate for drift, and there is no single renowned method that gives the best results in this matter [2]. Some of the above-mentioned aspects were inspirations for this study. Our goal was primarily to check the influence of these aspects on the accuracy of a sensor designed to test the ROM and shoulder proprioception (RSQ Motion sensor by RSQ Technologies LTD, Poznan, Poland) as well as the impact of the type of calibration and the test time on the accuracy of this sensor measurement.

IMU-based systems are a potential solution to provide cheap and reliable measurements in outpatient orthopedic clinics or in hospitals, but from the technical point of view, the measurement of the angles in human joints is not an easy task. The movement of a limb in a human joint is achieved by combining several different simple movements. For example, the extension movement of the knee joint in a plane parallel to the sagittal plane is the combination of the rolling, sliding, and rotation movements of the bones taking place on the surface of the joint. This aspect, as well as the method and location of sensor attachment and soft tissue artefacts, may contribute to the inaccuracy of a result indicated by an inertial sensor, e.g., during a range of motion test. Therefore, determining the accuracy of the RSQ Motion sensor in measuring anatomical angles requires further research. In extensive work published by Al-Amri et al. [4], it was shown that commercially available inertial systems, such as Xsens MVN BIOMECH, have fair-to-excellent reliability in measuring the angles of the hip, knee, and ankle joints in the sagittal plane during clinically relevant functional activities such as walking, squatting, or jumping. However, these systems should not be used interchangeably with optical motion capture systems, as the absolute values of angles differ between these systems and the offset is visible. In order to properly estimate the angular positions of body segments, IMU sensors intended for human motion tracking use kinematic representations of the body (virtual models) that include constraints specific to human joints. They model the human body as a parametrized kinematic chain with anatomical constraints such as joint angles in ranges corresponding to human joints that maintain connectivity between proper body segments [5]. For example, Cutti et al. [6] developed a study with the use of a shoulder model that used a set of assumptions that involved known functional and anatomical features. The upper limb was composed of rigid bodies representing the thorax, scapula, humerus, and forearm. The scapulothoracic and humerothoracic kinematics were measured with respect to six independent angles: protraction and retraction, medio-lateral rotation, anterior–posterior tilting, flexion and extension, abduction and adduction, and internal–external rotation. They used MT9B inertial sensors (Xsens Technologies, NL), which were assumed to have less than 1° of static accuracy and 3° of dynamic accuracy, expressed as root-mean-square error (RMSE) and measured for a single sensor [7].

The shoulder is the most mobile joint of the human body. It has the greatest range of motion and complicated mechanics; therefore, it is the best joint to test all sorts of movements and directions. Simultaneously, it is the most challenging joint for motion tracking with both OMC and IMU systems. However, from the clinical point of view, shoulder function can be efficiently tested with a set of simple movement tasks, such as flexion/extension, abduction/adduction, or internal/external rotation. These movements are used in clinical evaluations of the shoulder, e.g., after a surgical intervention, in the form of proprioception or ROM tests, but every device on the market to perform measurements of these movements should be carefully tested for accuracy. Sufficient accuracy of an IMU sensor depends on the application of the sensor (tested movement). For the measurement of joint angles in dynamic movements, their accuracy should be less than 5.0° so they can be comparable to optical motion capture systems [8], whereas in slow and precise movements (e.g., joint proprioception testing), it is recommended to use devices with accuracy values of less than 1° or even 0.1° [9,10].

Accuracy is usually measured in comparison to ground truth data obtained from an instrument treated as a “gold standard” (goniometer, robot arm, OMC, etc.) using the RMSE or correlation coefficients [2]. OMC systems are the gold standard used most frequently in similar studies and studies comparing the accuracy of sensors in performing simple, one-plane movements, especially for every new sensor entering the market. Hislop et al. [11] suggested that the comparison of the IMU sensors to the OMC systems should be preceded by studies with simple movements (e.g., provided by the robot arm), without the complexity of biomechanical modeling, so that the accuracy can be measured independently in all axes (roll, pitch, and yaw).

In summary, many factors contribute to the accuracy of the upper limb angle measurement performed with IMU sensors, such as the calibration methods and the fusion algorithms with body models used to translate sensor readings into understandable angles for practitioners [8,12]. For a comprehensive assessment of a sensor, both the device itself and the measurement method must be tested. Therefore, we developed a two-part study where part 1 deals with the validation of the RSQ Motion sensor reading of a highly precise industrial robot and part 2 deals with the validity and reliability of the RSQ Motion sensor in the measurement of the ROM of the shoulder. The primary objective of this part of the study was to examine the accuracy of the sensor compared to a highly precise industrial robot that imitates shoulder movements. Secondary objectives were aimed at measures potentially improving or affecting the accuracy, including whether and how the type of sensor calibration affects its accuracy and whether the time and magnitude of the tested angle have an impact on the accuracy of the sensor measurement. The hypothesis was that the accuracy of the tested sensor is very high (RMSE < 1°) but due to the drift effect that characterizes inertial sensors, the sensor error is greater for larger measured angles and greater for longer measurement times.

## 2. Materials and Methods

To calculate the accuracy of the sensor in measuring static angles, one RSQ Motion sensor (by RSQ Technologies LTD, Poznan, Poland) was placed on the handpiece of the in-line robot wrist (KUKA KR3 R540 AGILUS robot arm, by KUKA Robotics Corporation, Augsburg, Germany) so that the x axis of the sensor was parallel to the robot arm axis. The robot arm then performed 9 repetitions of 9 different angular positions (2.5°, 5°, 10°, 15°, 30°, 60°, 90°, 120°, and 160°), moving only one chosen robot segment, thus moving only in one chosen axis of the sensor. After reaching the set position, the robot waited 3 s and then returned to the starting position. This sequence of static angles was repeated in 11 series, with 30 s of waiting time between the series. All measurements were recorded in continuous measurement mode to check the influence of the test time on the accuracy of the sensor. Raw measurements from the RSQ Motion sensor were calculated to angular degrees, and the RMSE between the angular position of the robot and the sensor measurement was calculated. Subsequently, to check whether the sensor error is influenced by the magnitude of the measured angle or the test time, the Pearson correlation between the robot’s angular position and the sensor error was calculated.

The RMSE and the Pearson correlation were calculated for the sensor moving only in the roll and pitch axes, which corresponded to the axes used during the shoulder ROM testing (see Table 1 and the description in Section 2.1). Measurements from the RSQ Motion sensor were calculated to angular degrees using two different calculation methods as the angle between the Earth gravity vector and the sensor’s coordinate frame (as it took place in shoulder flexion or abduction) and as the change in the sensor’s orientation in the roll axis from the starting position (as it took place in the shoulder internal/external rotation test). Additionally, all of the calculations were performed with the sensor calibrated in two ways. First, based only on accelerometer readings (marked as ‘acc’) and, second, based on accelerometer and gyroscope readings (‘acc + gyro’). Details regarding the RSQ Motion sensor and the industrial robot used in this study, as well as the experimental setup, are described in the following sections.

### 2.1. RSQ Motion Sensor

The RSQ Motion sensor (by RSQ Technologies LTD, Poznan, Poland) is part of the RSQ Motion system, which is designed for the measurement, diagnosis, analysis, and progress monitoring of patients before/after orthopedic treatment and patients undergoing rehabilitation as well as for sport monitoring. This system is intended for use by clinicians, physiotherapists, researchers, and directly by patients at home to measure many parameters related to human body segment movement, e.g., range of motion or proprioception. The RSQ Motion sensor is composed of MEMS sensors: an accelerometer, a gyroscope, and a magnetometer. The sensor data are sent using the Bluetooth low-energy 2.4 GHz ISM band, as a digital electrical signal, to a hub (a USB device connected to a computer). Dedicated software processes the signal, filters it, and calculates the orientation of the motion sensor in the form of quaternions. Details regarding the construction of the sensor and the calculus for the measurement of orientation can be found in a previously published study [13].

According to the manufacturer’s recommendations, the RSQ Motion sensor should be placed on the arm of the tested person. In flexion or abduction, the angle between the arm and the trunk is measured as the angle between the Earth gravity vector (determined at the stage of sensor calibration prior to shoulder testing) and the coordinate frame of the sensor. The internal and external rotation of the shoulder are examined with the tested person in a horizontal position, and the RSQ Motion sensor is placed on the wrist (for the details of sensor placement, see part 2 of this study). Then, the angle of the internal/external rotation of the shoulder is measured as a change in the sensor’s orientation in the roll axis between the actual position and the starting position (Table 1). This approach allows the elimination of measurement error resulting from the deviation of the forearm in the transverse plane during the rotation test. In this study, the orientation of the RSQ Motion sensor was calculated from the raw values of the accelerometer and the gyroscope using the sensor fusion method and a complementary filter (Madgwick) [14]. The sensor’s magnetometer was turned off in order to avoid any interference with the iron housing of the robot, and the accelerometer’s trust value was set to 0.1.

The accuracy of the IMU sensors is, in general, highly dependent on the successful calibration process performed by the manufacturer in reference to the output of specialized high-precision equipment (e.g., the industrial robot) for which it is possible to precisely program a reference signal that should appear on the tested sensor at a given movement. Calibration is the process of determining the sensor signal coefficients (the cross-alignment, scale factor, and offset) so that the measurements from the inertial sensor are as close as possible to the reference signal against which the calibration is made. This means recording the measurement by the inertial sensor using high-precision equipment and then adjusting the coefficients of the inertial sensor in such a way that the signal (converted using the determined coefficients) is as close as possible to the known reference signal. These coefficients may be set with respect to the signal obtained from the accelerometer alone or with respect to the signals obtained simultaneously from the accelerometer and the gyroscope. Because of noise in the sensor data, the misalignment of the sensor axes, or inaccuracies in the robot’s position, the converted signal from the inertial sensor will never be exactly the same as the reference signal; however, the purpose of calibration is to minimize the sensor error, which is understood as the difference between the measured and reference signals.

**Figure 1:**
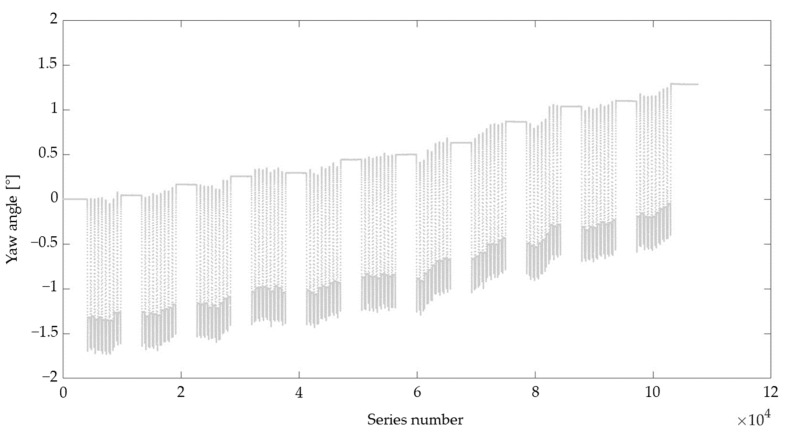
Readings in the yaw axis while the sensor was moving only in the roll axis (calibration based only on accelerometer readings).

**Figure 2:**
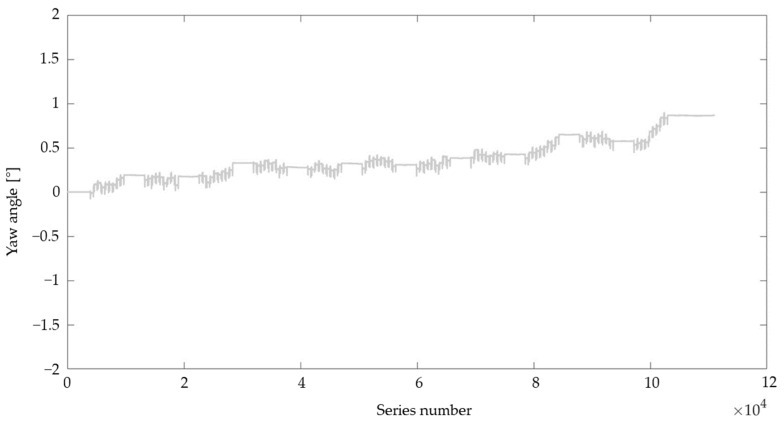
Readings in the yaw axis while the sensor was moving only in the roll axis (calibration based on accelerometer and gyroscope readings).

In this study, the RSQ Motion sensor was tested with two sets of configurations. First, the calibration parameters for each of the sensor axes were estimated based only on accelerometer readings and tests performed while the robot was in motion (referred to below as ‘roll (acc)’ and ‘pitch (acc)’). During the first pilot measurements, we noticed the unsatisfactory results of the sensor orientation readings in the yaw axis while the sensor was moving in the other axes. The neutral position was rising over time, and movements in the pitch or roll axes generated changes in the measurement of the yaw axis of about 1.5° (see the example in Figure 1). To minimize this effect, in the second approach the calibration parameters for each of the sensor axes were established based on the readings of the accelerometer and gyroscope (referred to below as ‘roll (acc + gyro)’ and ‘pitch (acc + gyro)’; Figure 2).

### 2.2. Industrial Robot

**Figure 3:**
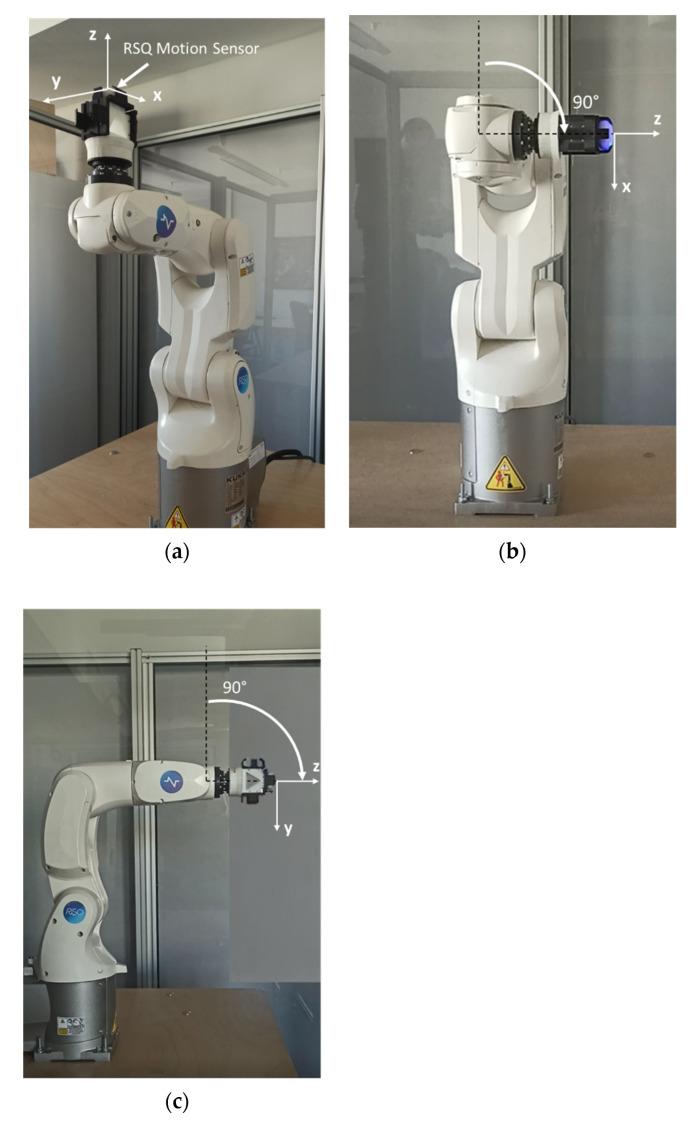
Sensor placement on the industrial robot arm: (**a**) sensor coordinate system in reference to the robot in the starting position, (**b**) an angle of 90° of rotation around the y axis (pitch movement) with a frontal view of the robot, (**c**) an angle of 90° of rotation around the x axis (roll movement) with a side view of the robot. Own source.

**Figure 4:**
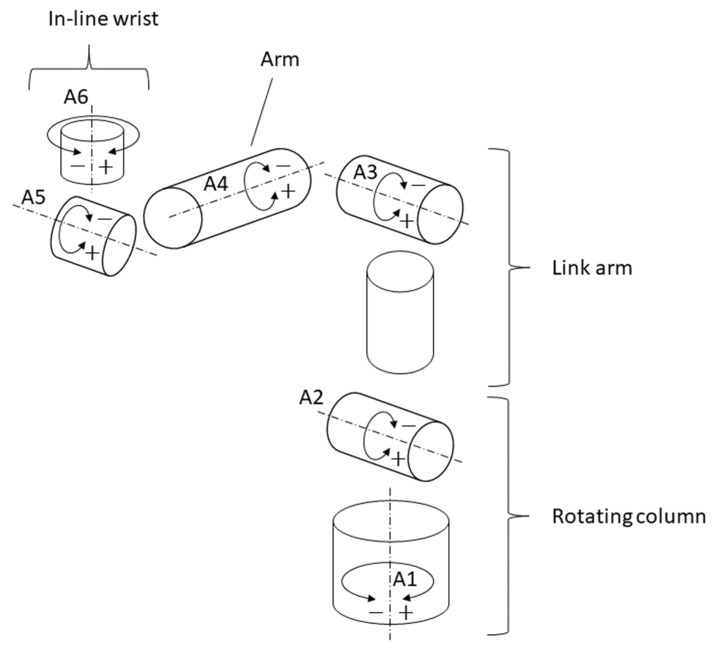
Schematic representation of the principal components of KUKA KR3 R540 AGILUS and its rotational axes. Own source.

KUKA KR3 R540 AGILUS is a highly precise lightweight industrial robot, designed for the production of small parts, with a maximum payload of 3 kg and a pose repeatability of ±0.02 mm [15]. An inertial motion sensor was placed on the handpiece of the in-line robot wrist so that the x axis of the sensor was parallel to robot arm axis A5, the y axis of the sensor was parallel to robot axis A4, and the z axis of the sensor was parallel to axis A6 (Figure 3 and Figure 4).

### 2.3. Experimental Setup

To validate the RSQ Motion sensor measurements compared to the KUKA KR3 R540 AGILUS industrial robot, nine different angular positions were tested (2.5°, 5°, 10°, 15°, 30°, 60°, 90°, 120°, and 160°) in reference to the starting position of the robot (Figure 3a). The experimental study and the measurement were designed to mimic shoulder movement in all axes: flexion, abduction, and rotations (Table 1). We chose the most important angular positions, from slight deviations (from 2.5° to 5°) up to functional ranges (with a maximum of 160°). The movements in the roll and pitch axes (the A4 and A5 axes of the robot), calculated with reference to the gravity vector, mimicked the flexion and abduction of the shoulder. Movement in the roll axis (A5), calculated as a change in the sensor’s orientation in the roll axis from the starting position, mimicked the internal and external rotation of the shoulder. Each position was recorded by the RSQ Motion sensor in continuous measurement mode with a sampling frequency of 114 Hz. The starting position of the robot used for this study is presented in Figure 3a and can be described as follows:

1. Rotation around the A1 axis: 0°;
2. Rotation around the A2 axis: 0°;
3. Rotation around the A3 axis: 90°;
4. Rotation around the A4 axis: 0°;
5. Rotation around the A5 axis: −90°;
6. Rotation around the A6 axis: 0°.

The starting position of the robot was selected so that each of the tested movements was performed by rotating only one robot segment while the other joints and segments were fixed. The rotation around the x axis of the sensor (roll movement) corresponded to the rotation by the given angle around the A5 axis of the robot. The rotation around the y axis (pitch movement) corresponded to the rotation by the given angle around the A4 axis of the robot.

For each of the angular positions, 9 repetitions were made in 11 series. The robot movement was performed at 30% of the robot’s maximum speed (approximately 108°/s). After reaching a given angular position, the robot waited for 3 s and then returned to the starting position. The waiting time between series was extended to 30 s. Translations between positions took less than 1 s. The total time of one series took around 40 s, and the total testing time of all angular positions in one axis took from 14 to 17 min, depending on the value of the tested angle.

### 2.4. Data Analysis

The raw measurements from the RSQ Motion sensor, in the form of quaternions, were calculated in angular degrees according to Madgwick [16]. After data were collected, MATLAB (The MathWorks, Inc., Natick, Massachusetts, United States) was used to analyze the signals. First, from all the samples collected by the RSQ Motion sensor, we extracted all the samples measured after the robot arm had reached a stable angular position. Each repetition of a given angular position was represented by about 305 samples measured by the sensor. Then, the root-mean-square error (RMSE) between the angular position of the robot and the sensor measurement was calculated. The RMSEs were analyzed separately for all tested angles (irrespective of the test series) and all series of tests (irrespective of the tested angle; Table 2).

The Pearson correlation was calculated to check whether the sensor error was influenced by the magnitude of the measured angle or the test time (understood as the test series). We evaluated the correlations between the angle set on the robot and the sensor error (named ‘robot angle vs. sensor error’ in Table 3) and between the test series and the sensor error (named ‘series vs. sensor error’ in Table 3). Sensor error was defined as the absolute difference between the angle set on the robot and the sensor reading. The absolute values of the Pearson correlation coefficient were interpreted as follows: r ≤ 0.35, weak correlation; 0.36 ≤ r ≤ 0.67, moderate correlation; 0.68 ≤ r < 0.9, strong correlation; r ≥ 0.9, very strong correlation [17].

## 3. Results

The RSQ Motion sensor appeared to be a very accurate sensor for measuring static angles (RMSE < 0.15° in all angles and series tested; Table 2). The RMSE for angles measured with the sensor calibrated using the second method (acc + gyro) was slightly higher than for the sensor calibrated only based on accelerometer readings (acc), especially for angles higher than 15° (roll (acc) vs. roll (acc + gyro) and pitch (acc) vs. pitch (acc + gyro) in reference to robot angle; Table 2). The calculated accuracy was very similar, regardless of the method used to calculate the final angle (see the RMSE values for angles calculated with reference to the gravity vector vs. angles calculated with reference to the sensor’s orientation in the starting position for the movements in the same axis; Table 2).

We found a moderate-to-strong correlation between the sensor error and the magnitude of the measured angle but only for the sensor calibrated with the gyroscope and accelerometer readings (a moderate correlation for roll (acc + gyro), with *r* = 0.58 and *p* < 0.001, regardless of the angle calculation method, and a strong correlation for pitch (acc + gyro), with *r* = 0.73 and *p* < 0.001; Table 3). There was only a weak correlation between the sensor error and the test time expressed as the test series (*r* from 0.12 to 0.35, *p* < 0.001), with exception of pitch testing (acc) (*r* = 0.51, *p* < 0.001). All the tested correlations were statistically significant (*p* < 0.001).

## 4. Discussion

This study examined, for the first time, the accuracy of the RSQ Motion sensor with the use of a high-precision industrial robot. The data provided in this study describe the static accuracy of the sensor. We have shown that a calibration performed with only the use of accelerometer readings is sufficient to achieve high accuracy in measuring the angles of simple one-plane movements in both methods of measurement, that is, with reference to the Earth gravity vector or the sensor’s orientation in the starting position. The accuracy was at the same level throughout the study (RMSE less than 0.1 and weak-to-moderate correlations for all ‘acc’ tests). The calibration performed using accelerometer and gyroscope readings (‘acc + gyro’ tests) slightly increased the sensor measurement error by about 0.05° compared to the ‘acc’ tests. Moreover, the second type of calibration was characterized by a moderate-to-strong correlation of the measurement error with the magnitude of the tested angle. The increase in the RSME was especially noticeable in the angle range of 30° to 120°. However, regardless of the type of calibration, the calculated accuracy was sufficient to test the basic movements of the shoulder.

The accuracy of the RSQ Motion sensor tested in this paper is comparable to the accuracy of the inertial motion capture systems available on the market and commonly used in medical applications, such as Xsens MTw Awinda (RMS for static angles in roll/pitch: 0.5°, yaw RMS: 1.0° [18]) or Noraxon Ultium Motion (RMS for static angles in roll: 0.025°, yaw RMS: 1.25° [19]). In slow and precise movements, such as shoulder proprioception testing, it is important to be aware of the accuracy of the device in particular axes and the calculation method contributing to the final measurements. In IMU sensors, the calculation of the angle based on raw sensor measurements may be divided, in general, into two methods, as mentioned previously, with origins in quaternions, but to measure precise movements it is recommended to use the most accurate axes (pitch and roll) [3]. This is the reason that in the RSQ Motion system, the external and internal rotation are measured differently from flexion and abduction in ROM or proprioception tests.

There are few limitations to this study. First, we did not perform an analysis of the sensor readings for the static sensor placed on the robot arm. Therefore, we did not investigate the sensor error resulting from the activity time and the surrounding noise. Similarly, the effect of the number of repetitions on the sensor error was not tested. We used 99 repetitions of every tested angle, as this was sufficient to obtain statistically significant results.

Considering the in vivo application of the sensors in a clinical setting, the second part of the investigation will be devoted to comparing the accuracy of the RSQ Motion sensor with a gold standard used in orthopedic research, such as goniometers or optical motion capture systems. It is expected that the error of the IMU sensor will be larger while testing on a human subject because measuring anatomical angles is not as simple as measuring angles for movements taking place in one plane or axis, as was mentioned in the introduction of this study. In future studies, it is planned to use more than one RSQ Motion sensor to study more complex motor activities, such as gait or sports activities (for example, testing a throw in overhead sports). However, measuring the accuracy of one sensor was necessary to establish a point of reference for sensor accuracy for future research.

## Figures and Tables

**Table 1:** The method of measuring the angle using the RSQ Motion sensor in the examination of the range of motion or shoulder proprioception.

	Flexion	Abduction	Internal/External Rotation
The angle calculation method	The angle between the Earth gravity vector and the sensor’s coordinate frame	The angle between the Earth gravity vector and the sensor’s coordinate frame	Change in the sensor’s orientation in the roll axis
Sensor placement on the body	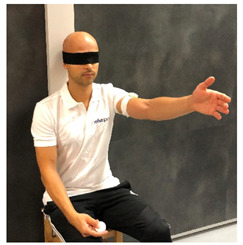	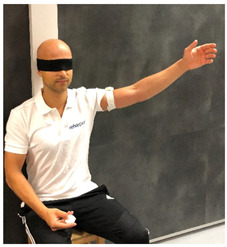	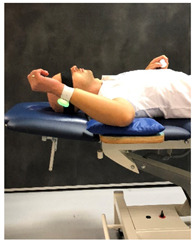

**Table 2:** Root-mean-square error (RMSE) between RSQ Motion sensor measurement and the position of the robot arm in relation to the robot angle and the test series.

Calculation Method	With Reference to the Earth Gravity Vector (Flexion and Abduction)				With Reference to the Sensor’s Orientation in the Starting Position (Internal/External Rotation)	
Axis (Calibration Method)	Roll (acc)	Roll (acc + gyro)	Pitch (acc)	Pitch (acc + gyro)	Roll (acc)	Roll (acc + gyro)
RMSE for all angles/series:	0.056	0.115	0.069	0.119	0.056	0.115
Robot angle:						
2.5°	0.064	0.070	0.067	0.057	0.064	0.071
5.0°	0.049	0.076	0.057	0.053	0.049	0.076
10.0°	0.058	0.057	0.062	0.062	0.058	0.057
15.0°	0.078	0.069	0.065	0.068	0.078	0.069
30.0°	0.050	0.093	0.051	0.104	0.050	0.093
60.0°	0.043	0.155	0.056	0.120	0.043	0.155
90.0°	0.053	0.147	0.050	0.153	0.053	0.147
120.0°	0.048	0.165	0.083	0.189	0.048	0.165
160.0°	0.058	0.134	0.111	0.164	0.057	0.134
Mean RMSE ± SD for all angles:	0.056 ± 0.01	0.107 ± 0.04	0.067 ± 0.018	0.108 ± 0.049	0.055 ± 0.01	0.108 ± 0.04
Test series:						
1	0.034	0.105	0.054	0.111	0.034	0.105
2	0.075	0.125	0.084	0.129	0.075	0.125
3	0.075	0.124	0.085	0.125	0.074	0.125
4	0.075	0.124	0.084	0.127	0.075	0.124
5	0.075	0.124	0.083	0.127	0.075	0.124
6	0.076	0.123	0.082	0.126	0.076	0.123
7	0.074	0.125	0.085	0.125	0.074	0.125
8	0.073	0.129	0.087	0.133	0.073	0.129
9	0.073	0.128	0.082	0.131	0.073	0.128
10	0.075	0.129	0.083	0.130	0.075	0.129
11	0.075	0.130	0.085	0.131	0.075	0.130
Mean RMSE ± SD for all series:	0.071 ± 0.012	0.124 ± 0.006	0.081 ± 0.009	0.127 ± 0.006	0.071 ± 0.012	0.124 ± 0.006

**Table 3:** Pearson’s correlation between sensor error and robot angle or time (series).

Calculation Method	With Reference to the Earth Gravity Vector (Flexion and Abduction)				With Reference to the Sensor’s Orientation in the Starting Position (Internal/External Rotation)	
Axis (Calibration Method)	Roll (acc)	Roll (acc + gyro)	Pitch (acc)	Pitch (acc + gyro)	Roll (acc)	Roll (acc + gyro)
	Robot angle vs. sensor error					
*p*	<0.001	<0.001	<0.001	<0.001	<0.001	<0.001
*r*	−0.030	0.579	0.338	0.726	−0.030	0.576
(95% CI)	(−0.033;−0.027)	(0.577;0.581)	(0.336;0.34)	(0.725;0.728)	(−0.033;−0.027)	(0.574;0.578)
	Series vs. sensor error					
*p*	<0.0001	<0.0001	<0.0001	<0.0001	<0.0001	<0.0001
*r*	0.351	0.125	0.507	0.116	0.352	0.125
(95% CI)	(0.348;0.353)	(0.123;0.128)	(0.505;0.509)	(0.113;0.119)	(0.349;0.354)	(0.122;0.128)

## Data Availability

Not applicable.
